# Co‐Designing Solutions to Improve Communication About Serious Illness During Hospitalisation

**DOI:** 10.1111/hex.70513

**Published:** 2025-11-27

**Authors:** Isabelle Caven, Melissa Frew, Jennifer Hyc, Warren Lewin, Jennifer Rosart, Amy Troup, Leanne Kim, Helen James, Senyo Agbeyaka, Aivan Chau, Richard Dunbar‐Yaffe, Karen Okrainec

**Affiliations:** ^1^ University Health Network Toronto Ontario Canada; ^2^ Department of Family and Community Medicine, Division of Palliative Care University of Toronto Toronto Ontario Canada; ^3^ Department of Medicine University of Toronto Toronto Ontario Canada; ^4^ Institute of Health Policy, Management and Evaluation, Dalla Lana School of Public Health University of Toronto Toronto Ontario Canada

**Keywords:** co‐design, patient experience, provider experience, serious illness communication, serious illness conversations

## Abstract

**Background:**

Serious illness conversations (SICs) that explore patient priorities are becoming increasingly important to high‐quality care for those with life‐limiting conditions admitted to general internal medicine wards. While patient‐cited barriers to SIC include a lack of understanding of complex medical terminology, expected illness course and life‐sustaining interventions, providers cite poor documentation and lack of training and support. For seriously ill patients to receive care that aligns with their values and goals, interventions that address these barriers need to be designed in consultation with patients and their providers and tailored to the context of their medical setting and broader health system.

**Methods:**

We report on the last phase of an overarching quality improvement project aimed at increasing documentation of SIC and improving patient/caregiver and provider experiences during SIC through co‐design. This phase was conducted on the general internal medicine wards at an academic teaching hospital network in Canada. Twenty‐five providers spanning various disciplines and departments participated in three co‐design workshops with 13 patients and caregivers between September and December 2023. Facilitated by experts in human‐centred design, the workshops sought to build on existing evidence and experience with known interventions to co‐produce solutions to SIC challenges. Patient, caregiver and provider priorities were established along with a set of design principles, guiding participants through solution ideation and refinement.

**Results:**

The collaboratively developed design principles (clear, compassionate, informed and reciprocal) guided the co‐production of solutions to improve in‐hospital SICs. Workshop attendees designed solutions that built on revising existing conversation guides, harnessing electronic medical record infrastructure to create collaborative advanced care planning notes, and supporting the role of a facilitator that could offer more structured and routine SIC during hospitalisation.

**Conclusions:**

Increasing knowledge of and access to SIC resources, such as patient conversation guides, advance care planning notes and establishment of Goals of Care Facilitators, were found in co‐design to be key solutions to address long‐standing barriers to engaging in high‐quality SIC and contribute to improving patient and provider experience and outcomes.

**Patient or Public Contribution:**

Patients and caregivers with lived experience were directly involved in the design sessions outlined in our study, setting priorities and developing solutions alongside healthcare providers and research staff.

## 1 Introduction

With an ageing population, serious illness conversations (SICs) are occurring more frequently on General Medicine inpatient wards. SICs encompass many topics, including those focused on cultivating patient/family prognostic awareness as a means to help them cope with their illness and those that delineate goal‐concordant care plans (Arnold et al. 2024). Patients and caregivers having difficulty accepting poor prognoses or understanding the limitations and complications of life‐sustaining treatments have been identified as barriers for providers conducting SICs [1]. In addition to barriers initiating these conversations, documentation of SICs remains low for patients admitted to the hospital [2]. The importance of moving from documenting solely patient wishes for life‐sustaining treatments to having more holistic conversations surrounding patient values and goals has positive benefits for both patients and providers [3]. Patients who had timely SICs reported improved quality of life and received more personalised end‐of‐life care [4]. For providers, there is some evidence to suggest that conducting SICs with patients can reduce moral distress and provider burnout [5].

Across North America, interventions have been implemented to train providers to improve the delivery and documentation of SICs [6, 7, 8, 9, 10, 11, 12, 13]. The Serious Illness Conversation Guide, developed by Ariadne Labs, is one part of an evidence‐based programme that includes training providers to deliver SICs using a structured approach and includes topics such as illness understanding, decision‐making preferences, patient goals and fears [14]. Implementation of the guide, as part of a broader system‐based intervention, with patients who had a diagnosis of cancer, resulted in earlier, more frequent SICs that had a greater focus on patient values, goals and illness understanding [15]. The guide is adaptable and to date has been piloted in various settings, such as dementia care, dialysis clinics and emergency departments [16, 17, 18]. At the academic institution where this project took place, an initiative to improve communication skills competency was recently established that adapted resources from Ariadne Labs and VitalTalk, another known SIC skills training programme backed by evidence [19], for local use. VitalTalk adds additional focus by teaching practical and concrete communication skills that further support providers to navigate the emotional complexity inherent to these conversations. These skills, focused on responding to emotion empathically, in our experience, allow providers to more confidently support patients and families and progress more comfortably through SICs. While newly available at some hospitals, including our own, communication skills training is not mandated for providers and therefore uptake is variable.

Healthcare providers on general internal medicine (GIM) wards care for an increasing number of clinically complex patient populations [20], and in‐hospital SICs are often needed to guide care decisions that are aligned to patient values and wishes [4]; however, there is little research exploring the feasibility of SIC interventions on GIM wards. To understand the state of SICs from the perspective of both GIM patients and providers, Phase 1 of our quality improvement (QI) project identified that many providers across interprofessional teams engaged in SICs with inpatients, though less than 50% had documentation of SICs, and patients identified limited recall of having had them [21]. Patients' and providers' experience with SIC alike were negatively impacted by siloed care communication within the hospital and between hospital wards and outpatient clinics.

### 1.1 Objectives

The objectives of the co‐design phase were to create health system solutions to improve the patient, family and provider experience and documentation of SICs on GIM wards by aligning knowledge gathered from our previous QI interviews and surveys of patients, their caregivers and providers, and informed by existing tools and resources.

## 2 Methods

### 2.1 Project Design

Co‐design, a human‐centred design approach, incorporates the experiences of both those delivering and receiving care and is a collaborative, flexible approach to directly improve in‐hospital SICs [22]. This methodology helps build a deep understanding of end‐user needs to inform solutions and interventions, placing people at the centre while also looking at the challenges that impact SIC from a system level [23]. Bringing together patients and multidisciplinary providers allowed for an in‐depth exploration of the process of the current state and needs for conducting SICs in the inpatient GIM setting in the second, co‐design phase.

### 2.2 Project Setting and Participants

**Figure 1:**
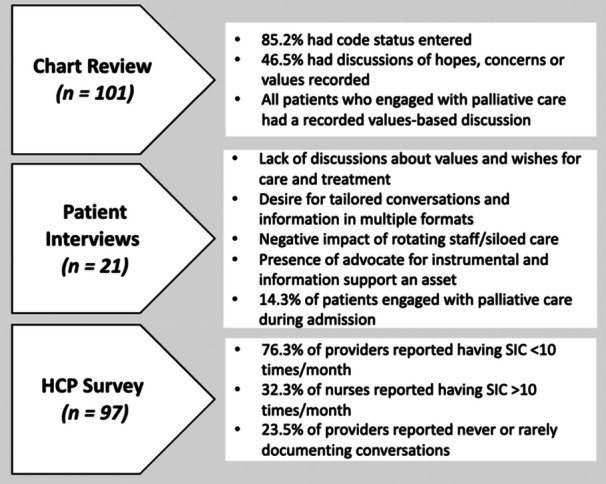
Summary of previous quality improvement work.

**Figure 2:**
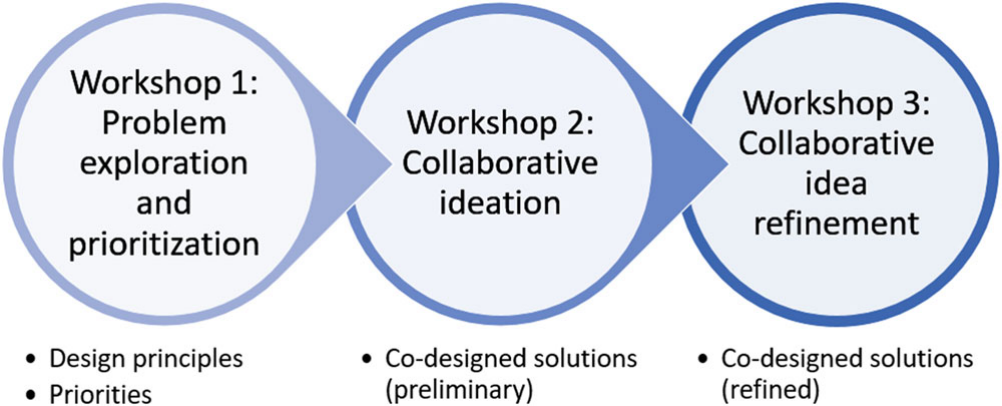
Co‐design workshop overview.

Findings from Phase 1 (healthcare provider surveys, chart review and semi‐structured interviews with patients and caregivers) provided the foundational knowledge for the co‐design process and are summarised in Figure 1 [21]. While the prevalence of SIC reported by providers was high, there were low rates of documentation of values‐based discussions, a patient‐identified desire for more SICs focused on values and goal‐based care, and how care siloes led to unmet communication needs. Only 14.3% of patients interviewed in Phase 1 had palliative care involvement during their stay, though other healthcare providers may have facilitated SIC conversations. Provider‐level findings highlight the multidisciplinary nature of SICs. The academic institution where our project took place has access to SIC resources, including tools aiding patients and providers to know what to discuss, communication skills e‐modules, VitalTalk‐powered simulation workshop training for providers to build and hone skills, and guidance for SIC documentation best practices (available at theconversationlab.org and AriadneLabs.org). The previous QI findings and existing resources guided three co‐design workshops hosted between September and December 2023 (Figure 2).

Patients, caregivers, physicians, nurses and allied health professionals (social workers, occupational therapists and physiotherapists) at two different hospitals, within a large academic teaching hospital network in a large urban centre in Canada, were invited to participate in the co‐design workshops. Patients/caregivers were recruited through flyers posted around two hospital sites and through advertising through the hospital's patient partners programme. Provider attendees were recruited through internal hospital list‐servs, by contacting providers who previously participated in the QI Phase 1 study, as well as those deemed to be good communicators and/or having previously expressed interest in the topic, and professional networks of the project team. All participants were invited to subsequent workshops, but could choose not to attend. Convenience sampling was used to recruit healthcare providers working in GIM, oncology, geriatrics and palliative care. Convenience sampling can potentially limit the generalisability of findings; however, iterative participant recruitment allowed us to target recruitment to address gaps in perspectives that arose at workshops (e.g., inviting a spiritual care provider to workshops following discussions of the importance of spiritual care as part of SICs).

Participants were offered $50 gift card honorariums. In addition to the recruited attendees, the interprofessional project team (spanning physicians, social work and nursing) attended all workshops, as well as research assistants. This project was deemed to be a QI initiative and was approved with a waiver of consent by the hospital's Research Ethics Board. All participants were provided an information letter with an overview of the project, the limits to confidentiality, and information that they could withdraw their participation at any time.

### 2.3 Data Collection

Trained facilitators in human‐centred design and healthcare engagement conducted the workshops. Prior QI findings (provider survey, EMR chart review and interview themes) were summarised and shared with all participants to help them understand the current state of SICs and available tools and resources across the hospital sites. Participants were led through a discussion about how the findings from data collection aligned or diverged from their own experiences and what they felt was most important in improving the quality of SICs from the patient and provider perspectives. An empathy mapping exercise was used to build empathy with key users who are engaged in SICs. Empathy mapping helped participants focus on what they wanted to feel, hear, see and do during an ideal SIC, as the topic can come with high emotion [24]. These prioritisation exercises were conducted during the first workshop to help develop our guiding design principles, develop cognitive empathy for the target end users of SIC [25], and align on the most impactful issues that could be solved for in the following two workshops [26]. Patient and provider priorities before, during and after SICs were established after Workshop 1. A set of design principles was generated that helped guide the overarching objectives for Workshops 2 and 3 (see Table 1), ensuring insights that emerged informed future design solutions and reflected the voices of end users (Cronholm et al. 2018).

**Table 1:** Attendees present at each workshop by role.

	Patient/Caregiver	Physician	Nursing	Social work	Spiritual care
Workshop 1	7	7 (5)*	3 (1)		
Workshop 2	3	3 (2)	1 (1)	1	
Workshop 3	8	8 (3)	8	2 (1)	1

* Numbers in brackets indicate the interdisciplinary project team members who are included in the total count and actively contributed to the workshops.

The facilitators generated ‘brainstorm questions’ before Workshop 2 to help guide participants through ideation and refinement of solutions. Workshop 2 generated patient‐centred ideas to address the prioritised problem areas, and Workshop 3 focused on refining these interventions, with provider input on feasibility and implementation challenges. Visual ‘sticky’ notes were used by participants to capture thoughts, experiences and ideas in response to prompts given by the co‐design facilitators [27]. An overview of the co‐design workshops is presented in Figure 2.

### 2.4 Analysis

At each workshop, members of the project team, summarising opportunities and barriers to improving SICs within the hospital network, took anonymous, detailed field notes. Following the workshops, experienced workshop facilitators synthesised field notes and sticky notes from the workshop activities. A debrief meeting was held after each workshop, where the synthesised findings were discussed and validated with the interdisciplinary project team. This iterative and cumulative process helped to refine the focus and activities of each subsequent workshop.

## 3 Results

Twenty‐five providers participated across three workshops, including 11 nurses, 11 physicians, 2 social workers and 1 spiritual care practitioner (see breakdown of workshop attendees in Table 1). Thirteen patients/caregivers participated in the co‐design process. Thirty four percent of participants attended more than one workshop. The outputs of each co‐design workshop are presented below, with Workshop 1 addressing design principles and priorities, and Workshops 2 and 3 focused on co‐designing and refining solutions that could later be implemented within the institution.

### 3.1 Co‐Design Outputs

#### 3.1.1 Workshop 1

The first co‐design workshop led with a set of design principles to guide what was collaboratively agreed upon to be necessary components for high‐quality SICs (Table 2). Key opportunity statements (derived from the synthesis of interviews conducted by the research team) were shared with each participant to individually provide their input, followed by a group discussion to collate and collaborate on insights. Dot voting was then used to identify priorities, which is a technique whereby participants are provided with stickers to mark ideas, opportunities or challenges to note relevance or importance (Table 3). Following the workshop, designers collated all responses and priorities from each room and conducted a thematic analysis. Empathy mapping informed the guiding principles that were refined with the project team and shared back in Workshop 2 for feedback from participants (Table 3).

**Table 2:** Co‐created design principles addressing key elements of serious illness conversations.

Principle	Definition	Impact
Clear	It can be hard for patients and families to understand the real meaning and implications of terms like ‘values’ and ‘palliative care’. When asking questions or providing answers, language must be simple and descriptive so that people can understand what it practically means for them and their families.	Clear questions and answers and the use of simple and descriptive language will ensure that people can understand what is being asked of and shared with them and their families.
Compassionate	Patients want to feel safe in SICs to talk about what they fear and to ask and answer questions using the words and language that feel most comfortable for them. They want to engage with a provider who is focused on them, doesn't appear to be rushed and prioritises their emotional comfort.	Patients who feel safe and comfortable will feel more able to share their fears and concerns with their provider.
Informed	Conversations will be most productive when both providers and patients have the information they need. For patients, this means knowing what SICs are and how to participate in them. For providers, this means knowing enough about a patient to lead a conversation that is clinically comprehensive and also tailored to their needs.	Patients who feel informed in advance of a conversation will have time to reflect and prepare, allowing them to be clear and concise during a conversation. Providers who are informed in advance about a patient will know who should be in the room, be prepared for possible questions and be able to offer clear guidance that feels personal.
Reciprocal	SICs should be two‐way conversations, in which there is time for patients, families and providers to ask questions and share what is most important to them. Patients and families want to feel like they are a part of the conversation, not the subject of the conversation.	Reciprocal conversations inherently make space for open questions and answers, thereby ensuring that patients have space to get the answers they need and providers are able to clearly share the information that is important.

**Table 3:** Priorities for addressing challenges with serious illness communication.

Theme	Dot votes received
Patient/Caregiver Reflection Before SICs Taking Place	22
Patient/Caregiver Access to Record of Conversations and Relevant Decisions Made	16
Opportunities for Patients/Caregivers to Reflect and Ask Questions After Conversations	10
Sharing of Conversation Notes Within the Circle of Care	16
Supporting Clinician Confidence in Conducting Conversations	15
Providing Structure for Conversations	25
Supporting Providers to Tailor Conversations to Individual Patient Needs/Preferences	17

#### 3.1.2 Workshop 2

Guided by the design principles and priorities established during Workshop 1, potential interventions were co‐created by patients and providers to improve the quality of SICs. Existing tools and resources within the hospital were highlighted and used as probes for discussion. From the provider perspective, the establishment of supportive teams, integrated electronic documentation and educational resources for providers engaging in SICs was explored. The interventions discussed during Workshop 2 were narrowed into two categories during synthesis: Patient Conversation Supports (comprising a summary report and conversation guide) and a Goals of Care Team.

#### 3.1.3 Workshop 3

During Workshop 3, the ideas established in Workshop 2 were refined, with emphasis on how the interventions would practically function for patients and providers within hospital settings. Systemic barriers and enablers were collaboratively explored, establishing a shared understanding of what next steps were required to build towards a new state where the co‐designed interventions could be implemented and evaluated.

#### 3.1.4 SICs Journey Map

**Figure 3:**
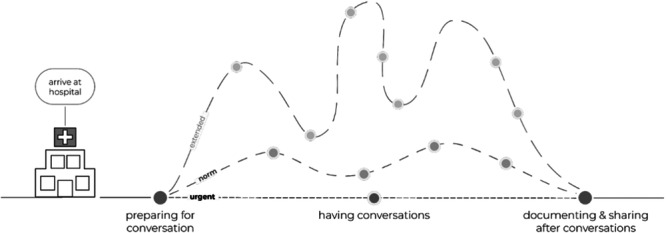
Talking about a serious illness journey map.

To help illustrate the nature of SIC and timing for co‐design interventions, an in‐hospital journey map was created by the workshop facilitators (Figure 3). Patients reported wanting to feel prepared before having SICs by having the information they needed and by understanding the rationale for having these conversations. Patients with a serious illness are therefore provided resources to help them understand their illness, the impact it may have on them over time and recommendations on how they can prepare for these iterative conversations moving forward. Providers wanted support and guidance from providers trained in leading SICs.

SICs are iterative and can occur multiple times in a patient's in‐hospital journey in response to changes to their clinical trajectory [28]. At less urgent points throughout the hospitalisation, conversations between patients and providers make space for building better prognostic awareness and discussing preferences and values that will allow patients to make informed decisions. Finally, documentation and sharing of conversations and next steps through a single record are done collaboratively and transparently to ensure that the patients' values are reflected accurately.

As a final step, three overarching opportunities were generated that address the design principles and priorities established in Workshop 1 and are described in further detail below.

##### 3.1.4.1 Leveraging Existing Patient Guides

The first co‐designed solution related to refining existing patient‐facing resources to better meet the needs and experiences of patients admitted to the GIM service that would benefit from having SICs. Patients and caregivers expressed a desire to be better prepared to engage in SICs before they occur. They also described a lack of familiarity with the typical language used during these conversations, which underscored the need for a resource to help them develop and appreciate a common language to address and ask questions about key serious illness topics. The strengths and weaknesses of an existing patient/family‐facing resource, adapted locally from Ariadne Labs' ‘pre‐visit letter’ (Bernacki et al. 2015) but not yet used widely at our institution, were discussed at the workshops. The guide encourages reflection on key SIC topics before speaking with a clinician about them. Leveraging the guide included co‐designed modifications to better meet the needs of all involved and included adding a section specifying which providers to contact to address questions with, creating space to document personal thoughts/questions, and adding questions specifically addressing treatment planning, as well as ensuring more patients have access to the guide (i.e., physical copies distributed on the ward as appropriate).

##### 3.1.4.2 Collaborative ACP Notes

The second co‐designed solution related to how SICs are documented and shared between both patients and providers. Findings from the EMR chart review identified low documentation of values‐based discussions and patients/caregivers who were interviewed expressed a desire to receive a copy of these conversations in various formats to support their recall and reflection (summary in Figure 1) (Caven et al. 2024 [under review]). The EMR at our hospital network, EPIC, allows providers to uniquely save relevant documented parts of an SIC into a unique Advance Care Planning (ACP) section of the patient's chart. For clarity, this EMR has the capacity to house all SICs in one section of the patient's chart, making it easy to know where and how to retrieve them when in‐the‐moment decision‐making is needed. Unfortunately, discussions during co‐design identified limited provider awareness of this feature. The co‐design process also identified a need for SIC documentation to be collaborative across interdisciplinary team members, increasing visibility of complex discussion topics across specialities and alignment of goals between key stakeholders, which in an academic setting is ever‐changing. Co‐design led some to advocate for institution‐wide education to bring awareness to the existing documentation tools, bolstering visibility across teams and sites that could help to minimise the perception of siloed care. Importantly, prior outpatient/clinic ACP documentation that is readily visible to the inpatient team could support in‐hospital iterative SIC, building on prior patient/family wishes.

##### 3.1.4.3 Barriers and Facilitators

At our institution, patients have access to their medical information (i.e., appointments and lab results) through an online portal. Having the ACP notes visible to patients through the portal was described by patient/caregiver participants as helpful for reinforcing a shared understanding of SICs and their outcomes. However, patients and caregivers alerted the team to the complex decision‐making issues that needed to be addressed and agreed upon before making these notes readily available to them. These included first knowing patient preferences towards knowing prognostic information and details about what to expect as the illness may progress over time. From a provider lens, there was concern over what does and does not routinely need to be communicated via a shared note with patients.

##### 3.1.4.4 Goals of Care Facilitator

**Figure 4:**
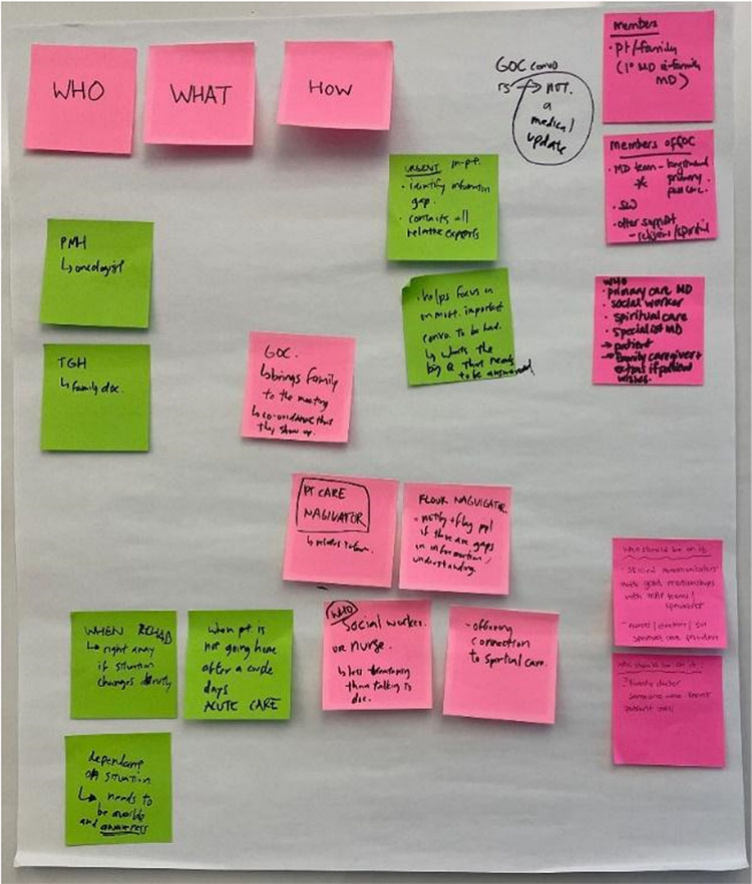
Sticky notes from Workshop 3: Barriers and Facilitators.

The final co‐designed solution was the introduction of a GOC facilitator, who would support patients/caregivers and medical teams through their entire hospital journey with respect to preparing for, having and documenting SICs. The need for this unique role came about because of local clinical time, competency and role description limitations that were identified. This role would be embedded into an existing team or ward and prioritise working collaboratively to address unmet SIC needs from admission through discharge and into follow‐up post‐discharge. Moreover, the role would serve to build knowledge and skill for the broader team, which would serve to eliminate the need for such a role over time. The role would ideally be filled by an existing team clinician and would require them to: (1) have dedicated time in this role and (2) have trained with evidence‐based SIC programmes like Ariadne Labs and VitalTalk. Sample role tasks could include leading evidence‐based SIC skills training, championing SIC implementation strategies on the ward, such as helping teams to identify patients that could benefit from timely SICs, and assisting the team in knowing how and where to document the conversation in the appropriate section of the EMR. Figure 4 represents the sticky notes that were generated through discussions about a GOC facilitator.

While some health systems utilise a team social worker or consulting palliative care team to address some of the above tasks, members present during our co‐design workshops highlighted the importance of SICs being done by all members of the care team. The facilitator role would additionally address communication challenges identified by patients. As identified through interviews and from the patients/caregivers at co‐design, SICs can be a nebulous term. The facilitator could serve an important advocacy role and help prepare families for these conversations, what to expect, and allow them to reflect on individual values/worries ahead of conversations and assist them in documenting these conversations. The facilitator could also help to bring in providers that patients/families had built relationships with outside of their inpatient admission—like a family doctor or oncologist.

The facilitator could address key challenges that providers experience in conducting SICs. Provider participants at co‐design emphasised that they still wanted to have SICs themselves. Physician attendees stressed that they needed to remain responsible to their patients for sharing the important medical information that underpins these conversations, and patients echoed the desire to have their physicians participate in SICs with them. For inpatient providers, they are balancing addressing the acute medical issues that brought the patient to the hospital, while also communicating with patients/families about a myriad of challenging topics—such as a worsening of a diagnosis, a shortened prognosis or a patient who is dying. This facilitator would therefore support providers by dealing with the logistical planning of conversations and preparing patients/families.

## 4 Discussion

For seriously ill patients admitted to GIM units, conversations encompassing goals and values are commonplace and present a critical opportunity to align care to their wishes and preferences. Throughout their hospital admission, patients and caregivers may be learning about a new life‐limiting diagnosis, altered illness trajectory or shortened prognosis. Conversations that explore individual patient values, goals and preferences are not only needed to align medical care to an individual's needs but can contribute to reducing what has been described as the trauma of hospitalisation [29]. However, myriad barriers, from both the patient and provider lens, can prevent these conversations from occurring, being documented or impacting their fulsomeness. Through this project, patients and providers co‐produced a set of design principles for SIC solutions that should be clear, compassionate, reciprocal and informed. The co‐designed solutions included (1) refining and ways to increase uptake and engagement when using existing patient‐facing guides, (2) collaborative ACP notes, and (3) implementing a GOC facilitator. Cutting across all three co‐designed solutions was the importance of learning about patient values and shifting conversations from ‘What's the matter with you?’ to ‘What matters to you?’ [30].

The first co‐designed solution was to modify existing patient‐facing guides and increase patient access, which addresses several priorities. For patients, having access to a guide that could help them understand key aspects of SICs and provide them opportunities for reflection before a conversation was an important priority. Unplanned admission to the hospital is an overwhelming, challenging experience, and patients/families may not even know what questions to ask to fill their gaps in understanding the acute situation. Increasing access to a patient‐facing guide can empower patients/families to know what questions to ask to understand their illness and what to expect during hospitalisation and beyond. Co‐designed modifications to the existing guide available within the hospital network will serve to address identified priorities and improve engagement, use and impact of the tool (i.e., space to write down reflections or notes from conversations). Workbook‐style adaptations to existing Serious Illness Conversation Guides have been previously created, and interviews of patients/caregivers who used the workbooks identified it as a useful, safe, acceptable and easy‐to‐use tool [31]. Importantly, presentation of the workbook from a provider with whom the patient had built rapport and explained the need for the tool.

The second co‐designed intervention was collaborative ACP documentation. This solution addresses several priorities, including access to notes within the circle of care and supporting providers in learning about patients to conduct conversations. Providers at co‐design workshops expressed challenges in knowing what previous SICs had taken place before or during the hospitalisation by other providers. Timely, collaborative and seamless documentation of key components to high‐quality SICs that is easily accessible to a care team can support providers in knowing up‐to‐date information that is critical to discussing and providing goal‐concordant care. Importantly, it can also highlight patient and family gaps in understanding or unmet needs that can be addressed across the interprofessional team. Identified in the literature is the lack of an easily retrievable location for documentation of ACP in the EMR, or overlapping, inconsistent documentation [32]. Leveraging our health system's EMR ACP section and tools is a vital avenue to increasing support for seamless, high‐quality serious illness care and should be the focus of future research. Existing research on the ‘Patient Values Tab’ in the EMR identified varied levels of access by interprofessional team members (i.e., chaplains, advance practice providers, nurses and attending physicians) [33], and more work is needed to ascertain how EMR documentation can support interprofessional care collaboration. However, increasing hospital‐level awareness of EMR ACP tools has been shown to increase documentation rates [8]. Given the high number of providers that care for patients admitted to GIM, and the frequent staff turnover that is present in an academic institution, more consistent documentation can support members of the circle of care in learning more about patients/families to build the rapport that is needed to have quality, patient‐centred conversations. Documentation, however, does not replace the patient–provider interactions that are needed to have high‐quality communication and can be used to support and accelerate the rapport‐building needed for SICs [34].

The final co‐designed intervention, a GOC facilitator, addresses several of the identified priorities for improving serious illness care. The facilitator can serve as an important advocate and support patient/caregiver pre‐conversation reflection and preparedness, post‐conversation reflection and questions, communication within the care team, support clinician confidence and communication training, and increasing frequency of SICs. Existing models of similar GOC facilitators, both lay‐person and clinical staff, can be built on to address specific needs as identified through co‐design. In studies exploring the role of a layperson GOC navigator, facilitators increased physician engagement and rapport building, while lack of time, limited stakeholder support, and patient‐level discomfort with the subject matter acted as barriers [35, 36]. While these barriers and facilitators align with our identified priorities, the co‐designed GOC facilitator was identified through co‐design as needing to be a healthcare professional. In another study, patients above the age of 65 enrolled in a primary care practice were randomised to a nurse navigator‐led ACP pathway or usual care. This ACP pathway led to an increased frequency of ACP discussions and their documentation in the EMR [37]. The GOC facilitator role is also intended to promote interprofessional collaboration, ease of planning conversations and supporting trainees to observe SICs. Other work has identified that training SIC ‘champions’ across clinical roles can support facilitation of inpatient SICs [38]; however, there is limited evidence that demonstrates how this champion role can address the co‐designed priorities (i.e., rapport building, reflection and awareness among the circle of care). Specific to the GIM setting, implementation of a facilitator would serve to reinforce the shared responsibility of SICs and not solely a palliative care consultation task. Increasing patient volumes and complexity may deprioritise SICs on the medicine wards; however, the GOC facilitator would serve to ensure these conversations took place, and all members of the care team can provide their input and expertise.

### 4.1 Future Directions

The co‐produced solutions require further exploration of their feasibility, scalability and sustainability within our academic hospital network before implementation. Since completion of the project, we have presented findings to key knowledge users (larger audiences of GIM physicians, nurses, social workers, trainees, etc.) to increase awareness and to understand how the proposed solutions could be integrated into their workflows, with no new barriers or challenges identified. Next steps include securing funding to pilot a GOC facilitator who would also integrate the provision of the modified patient and family workbook into their role. Changes to the ACP interface (across EMR and patient‐facing website) are also underway to support collaboration and reduce the risk of siloed care, particularly in the absence of a current GOC facilitator.

### 4.2 Limitations

These findings should be interpreted within the constraints present in the study design as a QI project and the feasibility challenges of implementing the co‐designed interventions outside a similar academic institution and health service. While the co‐design process allowed us to engage with key knowledge users and directly meet the SICs' needs in our academic hospital network, these findings may not be generalisable to other settings where SICs' resources and training are already implemented, or where there is a lack of interdisciplinary providers and trainees who conduct and collaborate on SICs and document using EMRs. In addition, the co‐designed solutions were framed within resource constraints present in our hospital system. For example, an identified barrier to SICs, through our work and other studies [39], is the lack of a private, quiet space to have discussions that are emotionally laden. We limited the outputs of the co‐design workshops to feasible, realistic solutions; however, system‐level challenges, like a lack of privacy, are important considerations for SICs. Lastly, through this project, the experiences of patients/caregivers were sought and grounded in the co‐design process. SICs can be an emotionally difficult experience for some patients/caregivers, and it is possible that unique perspectives were missed in the subset of patients/caregivers involved in this project who may not have felt comfortable discussing their experiences. Demographic diversity of participants was not tracked through co‐design, which limits the extent to which we understand the findings to be representative of the general population, especially from the patient and caregiver perspectives.

## 5 Conclusion

Communicating with patients and caregivers about their values, goals and preferences lays the foundation for providing holistic and patient‐centred care. Several serious illness communication tools and resources have been created to support higher quality and timely conversations to support goal‐concordant care and implemented across various clinical settings. However, to date, there has been limited exploration of how these tools and resources can practically support SICs for patients admitted to a GIM ward within a large multi‐site healthcare system. Informed by patient and provider‐level priorities, our co‐designed principles informed solutions which serve to support the preparation, documentation, collaboration and reflection that is needed for patients/caregivers and providers to have meaningful conversations to align inpatient medical care with what truly matters to each individual patient.

## Author Contributions

**Isabelle Caven:** conceptualisation, investigation, data curation, formal analysis, project administration, writing – original draft, writing – review and editing. **Melissa Frew:** conceptualisation, methodology, investigation, data curation, formal analysis, visualisation, writing – original draft, writing – review and editing. **Jennifer Hyc:** writing – original draft, writing – review and editing. **Warren Lewin:** conceptualisation, investigation, validation, writing – review and editing. **Jennifer Rosart:** conceptualisation, methodology, investigation, data curation, formal analysis, visualisation, supervision, writing – review and editing. **Amy Troup:** project administration, writing – review and editing. **Leanne Kim:** conceptualisation, validation, writing – review and editing. **Helen James:** conceptualisation, validation, writing – review and editing. **Senyo Agbeyaka:** validation, writing – review and editing. **Aivan Chau:** conceptualisation, validation, writing – review and editing. **Richard Dunbar‐Yaffe:** conceptualisation, methodology, investigation, validation, resources, funding acquisition, supervision, writing – review and editing. **Karen Okrainec:** conceptualisation, methodology, investigation, validation, resources, funding acquisition, supervision, project administration, writing – review and editing.

## Ethics Statement

This project received Research Ethics Board exemption from the University Health Network Research Ethics Board as it qualified as a quality improvement initiative (QI #22‐0489).

## Conflicts of Interest

The authors declare no conflicts of interest.

## Data Availability

Data sharing is not applicable to this article as no datasets were generated or analysed during the current study.
